# Robot-assisted Laparoscopic Retroperitoneal Lymph Node Dissection for Testicular and Upper Tract Urothelial Cancer—Surgical Technique and Outcomes of a Single-surgeon Series

**DOI:** 10.1016/j.euros.2025.03.015

**Published:** 2025-04-15

**Authors:** Marc A. Furrer, Benjamin C. Thomas

**Affiliations:** a Department of Urology, The University of Melbourne, Royal Melbourne Hospital, Parkville, Victoria, Australia; b Department of Urology, Solothurner Spitäler AG, Kantonsspital Olten and Bürgerspital Solothurn, University of Bern, Olten/Solothurn, Switzerland

**Keywords:** Robot-assisted surgery, Surgical procedure, Retroperitoneal lymph node dissection, Testicular cancers, Upper tract transitional cell carcinoma

## Abstract

**Background and objective:**

Retroperitoneal lymph node dissection (RPLND) is essential in managing testicular and upper urinary tract urothelial cancer (UTUC). While open RPLND remains the gold standard, robot-assisted RPLND (RA-RPLND) is gaining traction in selected cases. This study aims to describe the surgical technique and our experience with RA-RPLND, and to demonstrate the peri-and postoperative safety and efficacy of this approach for the treatment of testicular cancer and UTUC.

**Methods:**

We analyzed the data from a single-surgeon series of 96 patients (64 testicular cancer and 32 UTUC) who underwent RA-RPLND between 2016 and 2024. The procedures included left (*n* = 49), right (*n* = 31), and bilateral (*n* = 16) template dissection. Bilateral and unilateral templates were used for testicular cancer, while unilateral templates were applied to all UTUC cases involving high-grade disease in the kidney, proximal ureter, or midureter. Surgical indications, preoperative assessment, and postoperative care protocols are described. Baseline characteristics, peri- and postoperative data, and oncological outcomes were assessed. Complications were graded using the Clavien-Dindo classification.

**Key findings and limitations:**

Median length of stay was 1 (IQR 1–1) d for testicular cancer and 2.5 (IQR 2–4) d for UTUC patients. Open conversion occurred in two testicular cancer salvage cases. Major complications (Clavien-Dindo ≥3a) occurred in 9% (testicular cancer) and 13% (UTUC) of patients. Two patients died within 90 d after RA-RPLND for UTUC: one due to an acute myocardial infarction and the other due to progressive disease. Six patients (19%) with UTUC died due to progressive disease within a median follow-up of 38 (range 4–66) mo, whereas all patients with testicular cancer were still alive after a median follow-up of 46 (range 1–97) mo. Overall and cancer-specific survival rates at the end of follow-up were 78% and 69% in patients with UTUC, and 100% and 100% in patients with testicular cancer, respectively. No retroperitoneal recurrences occurred in either cohort until the end of follow-up. Limitations include the steep learning curve and nonreproducibility by surgeons without expertise in advanced robotic surgery.

**Conclusions and clinical implications:**

RA-RPLND remains a technically challenging operation, but is safe and effective in expert hands and should therefore be considered for selected patients in high-volume centers.

**Patient summary:**

In this study, we examined the outcomes after robot-assisted retroperitoneal lymph node dissection. We conclude that it is a safe and effective procedure for patients with testicular cancer and cancer of the renal pelvis and ureter when performed by experienced surgeons. Therefore, it can be a suitable choice for certain patients, depending on their individual circumstances. Patients should also be given detailed instructions about what to expect after surgery, including how to take care of themselves at home to promote recovery and stay safe after being discharged from the hospital.

## Take Home Message

Retroperitoneal lymph node dissection is essential in managing testicular and upper urinary tract urothelial cancer. While the open approach remains the gold standard, robot-assisted retroperitoneal lymph node dissection is gaining traction in selected cases. It remains a technically challenging operation, but is safe and effective in expert hands and should therefore be considered for select patients in high-volume centers.

## 1 Introduction

Retroperitoneal lymph node dissection (RPLND) forms an integral part of the multidisciplinary management of testicular and upper urinary tract urothelial cancer (UTUC).

In men aged 20–40 yr, testicular cancer is the most common malignancy, with 30% of these patients diagnosed with metastases at first diagnosis [1], [2]. Cisplatin-based chemotherapy regimens have revolutionized the treatment of testicular cancer, and consequently the role of surgery has changed. However, their combined role has resulted in overall survival rates of over 90%. While open RPLND (O-RPLND) remains the gold standard, robot-assisted RPLND (RA-RPLND) is increasingly being used in selective cases for stage 2 disease in both the primary and the postchemotherapy setting, with no absolute indications for a robot-assisted approach being considered. Postchemotherapy RPLND is known to be more challenging than primary RPLND due to the fibrosis associated with masses that have shrunken significantly after chemotherapy.

UTUC is a rare malignancy and accounts for 5–7% of all renal tumors and 5–10% of all urothelial tumors, peaking around the age of 70 yr [3], [4]. Renal pelvis tumors occur twice as commonly as ureteral tumors and are likely to have a higher stage with T3/T4 disease and a higher rate of lymph node metastases (10–35% vs 6–10%) at first diagnosis [3], [5], [6], [7], [8]. Lymph node metastases are found in 20–30% of patients undergoing radical nephroureterectomy [7]. Based on laterality and location of the primary tumor, UTUC follows the route of metastases to regional lymph nodes that typically precede the identification of visceral metastases [9].

Anatomically, lymphatic drainage differs between the left and right side. On the left, lymphatic channels feed into paraaortic, preaortic, and retroaortic lymph nodes, and on the right, these feed into paracaval, precaval, retrocaval, and interaortocaval lymph nodes. For the left midureter, the drainage is mostly into the paraaortic lymph nodes, whereas for the right midureter, lymphatic drainage is toward the paracaval and interaortocaval lymph nodes. The distal ureter is drained by pelvic (common, external, and internal iliac) lymph nodes on each side [10].

Lymph node dissection (LND) for UTUC has a beneficial impact on oncological outcomes by improving cancer-specific survival and lowering the risk for local recurrence, even in patients with clinically and pathologically negative lymph nodes [11], [12], [13], [14].

According to the current European Association of Urology guidelines, template-based LND should be offered to all patients scheduled for radical nephroureterectomy [15], [16]. Conversely, the National Comprehensive Cancer Network guidelines recommend template-based LND in patients with high-grade histology, large (>3–4 cm) primaries, or tumors with apparent parenchymal invasion only.

While the role and similar technique of RA-RPLND for testicular cancer have been described more recently [17], [18], [19], tremendous variation exists in surgical practices for performing LND in UTUC patients. Although templates for LND in UTUC patients have been reported previously [9], [20], reports of RA-RPLND for UTUC are emerging.

The aim of this article is to describe the surgical technique and our experience with RA-RPLND, and to demonstrate the peri- and postoperative safety and efficacy of this approach for the treatment of testicular cancer and UTUC.

## 2 Patients and methods

### 2.1 Patient population and data collection

We present prospectively collected data until the last follow-up of a consecutive single-surgeon series of 96 patients (testicular cancer, *n* = 64; UTUC, *n* = 32) who underwent RA-RPLND between 2016 and 2024. Complications were graded according to the Clavien-Dindo classification [21], [22], [23]. Ethics approval was obtained through Melbourne Health (QA2020046).

### 2.2 Indications and contraindications for RA-RPLND

There are no absolute indications for considering a robot-assisted approach. The relationship of the underlying disease is perhaps the most important aspect.

In all patients, the indication was made on an individual risk assessment and after shared decision-making with the patient. Table 1 provides a preoperative checklist to ensure a safe procedure.

**Table 1:** Preoperative checklist to ensure a safe procedure

Factors for consideration of robot-assisted RPLND		
1. Size of retroperitoneal mass or masses 2. Extent and distribution of retroperitoneal disease 3. Relationship of retroperitoneal disease to great vessels 4. Degree of size decrease after chemotherapy 5. Number of lines of chemotherapy preceding surgery		
Absolute contraindication for robot-assisted approach for testicular cancer		
1. Encasement of great vessels 2. Involvement of multiple viscera		
Advantages and disadvantages of the robot-assisted versus open approach		
Advantages	Disadvantages	

1. Magnification for nerve sparing 2. Smaller incisions 3. Decreased blood loss 4. Decreased bowel mobilization 5. Decreased analgesia requirements 6. Shorter length of stay and quicker recovery	1. Lack of approach options for vascular repair 2. Time required for conversion to open procedure in event of hemorrhage 3. Cost	
Absolute contraindication for the robot-assisted approach for upper tract urothelial cancer		
1. Invasive or large tumors (clinical stage T3/T4) 2. Lymph node or distant metastasis		
Robotic and special equipment required		
Robotic instruments	Sutures	Additional equipment

1. Fenestrated bipolar forceps 2. Monopolar scissors 3. Prograsp retractor 4. Large needle driver	“Rescue sutures” for vascular repair: 4/0 polypropylene (Prolene), 10 cm, with or without Hem-0-Lok applied	1. AirSeal insufflator 2. 30° or 0° scope

RPLND = retroperitoneal lymph node dissection.

The main indication was stage 2 nonseminomatous germ cell tumors with evidence of retroperitoneal lymph node involvement on imaging or if there is a residual mass in the retroperitoneum after chemotherapy (particularly for advanced testicular cancer). Furthermore, if a patient experiences a recurrence of cancer in the retroperitoneal lymph nodes, RPLND can be indicated as part of a salvage treatment strategy. Increasingly, RA-RPLND was utilized for stage 2 seminoma in a trial setting.

In certain cases, RPLND was indicated due to diagnostic uncertainty, such as inconclusive imaging or tumor marker levels, to obtain tissue for a histopathological examination and confirm the nature of the disease. In rare cases, RPLND may be indicated for fertility preservation. As such, RPLND can be performed in a nerve-sparing manner to reduce the risk of infertility associated with the procedure. In particular, we performed all RPLND procedures for testicular cancer for masses >1 cm (whether primary or after chemotherapy). With this regard, we used the mass length on abdominal imaging prior to chemotherapy for clinical N staging.

For UTUC, RPLND was performed for renal pelvis, proximal, or midureteral tumors with high-risk features such as high-grade cytology, high-grade disease on biopsy, suspicion of clinical T3 disease, or tumor size >3 cm.

Detailed information on the indications and contraindications, preoperative assessment, and postoperative instructions is provided in the Supplementary material.

### 2.3 Surgical technique

Herein, we describe the different templates (ie, unilateral left and right, and bilateral template dissection), stages of the procedure, and the surgical technique in brief. A detailed systematic description of all procedural steps is provided in the Supplementary material.

#### 2.3.1 Theatre and port setup

**Fig. 1:**
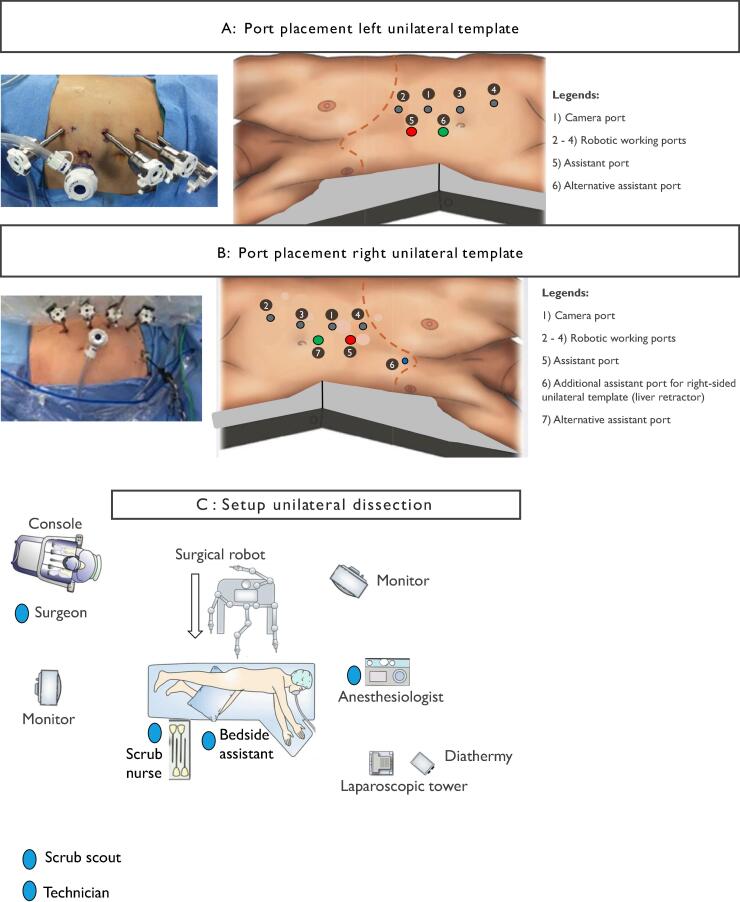
(A) Port placement left unilateral template. (B) Port placement right unilateral template. (C) Setup unilateral dissection.

**Fig. 2:**
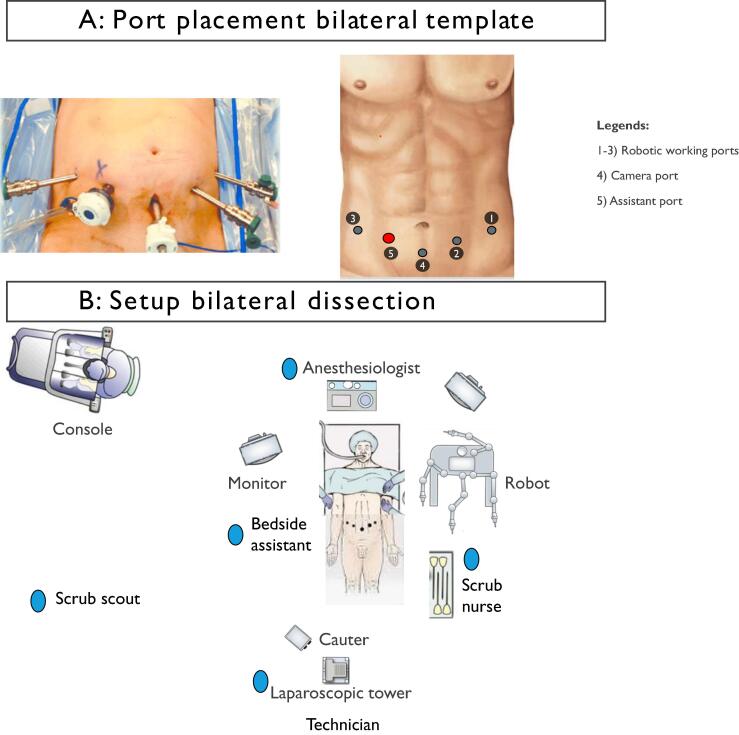
(A) Port placement bilateral template. (B) Setup bilateral dissection.

**Fig. 3:**
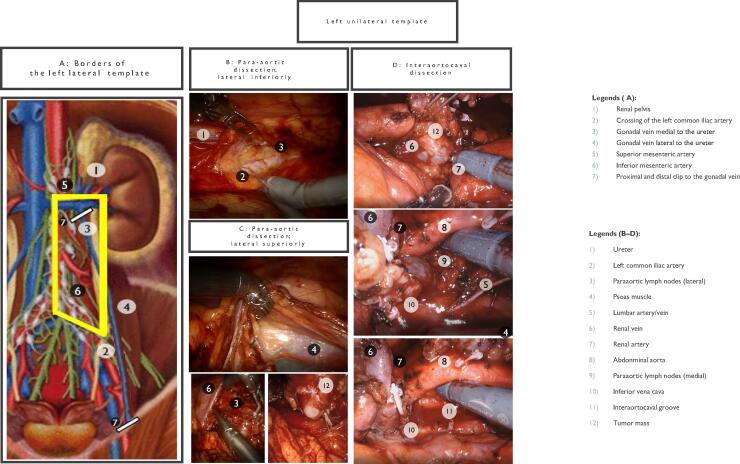
Left unilateral template. (A) Borders of the left lateral template. (B) Paraaortic dissection; lateral inferiorly. (C) Para-aortic dissection; lateral superiorly. (D) Interaortocaval dissection.

**Fig. 4:**
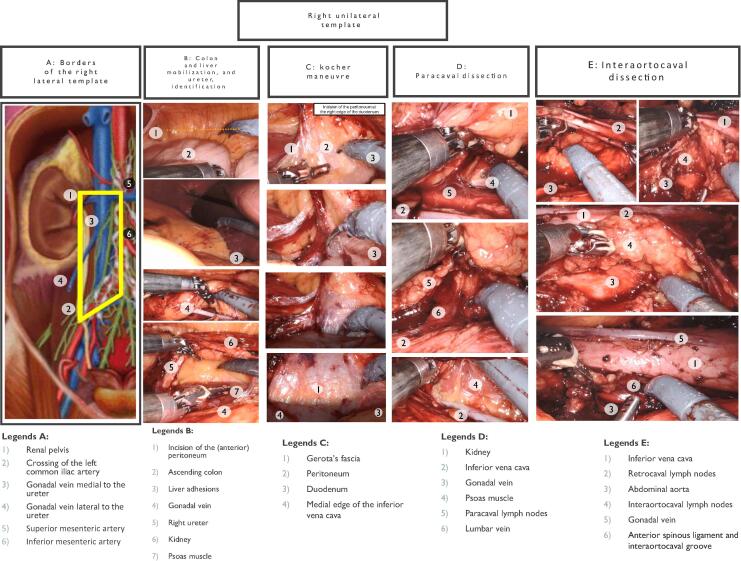
Right unilateral template. (A) Borders of the right lateral template. (B) Colon and liver mobilization, and ureter identification. (C) Kocher maneuver. (D) Paracaval dissection. (E) Interaortocaval dissection.

**Fig. 5:**
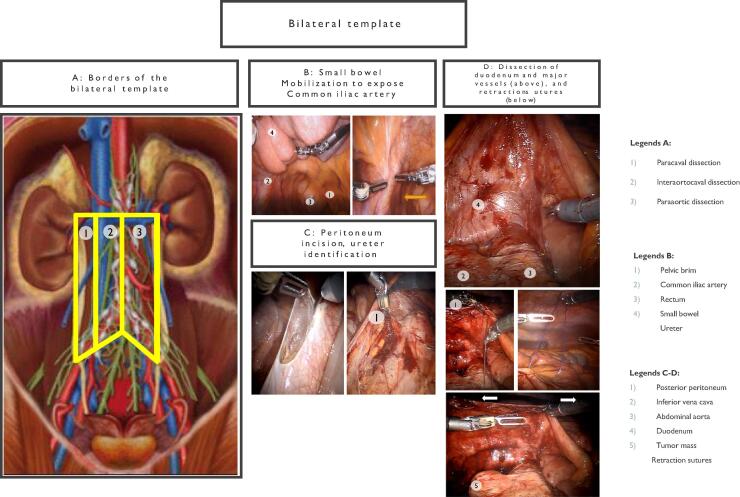
Bilateral template. (A) Borders of the bilateral template. (B) Small bowel mobilization to expose common iliac artery. (C) Peritoneum incision and ureter identification. (D) Dissection of duodenum and major vessels (above), and retraction sutures (below).

**Fig. 6:**
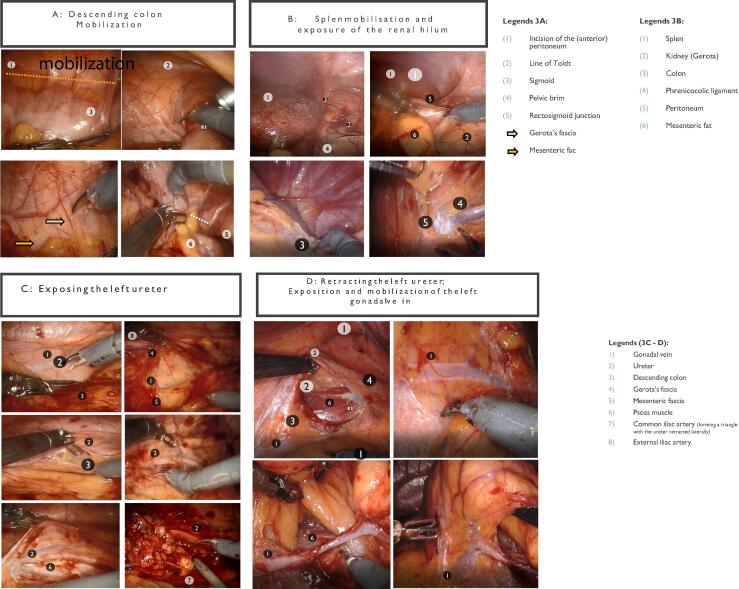
(A) Descending colon mobilization. (B) Spleen mobilization and exposure of the renal hilum. (C) Exposing the left ureter. (D) Retracting the left ureter; exposition and mobilization of the left gonadal vein.

**Fig. 7:**
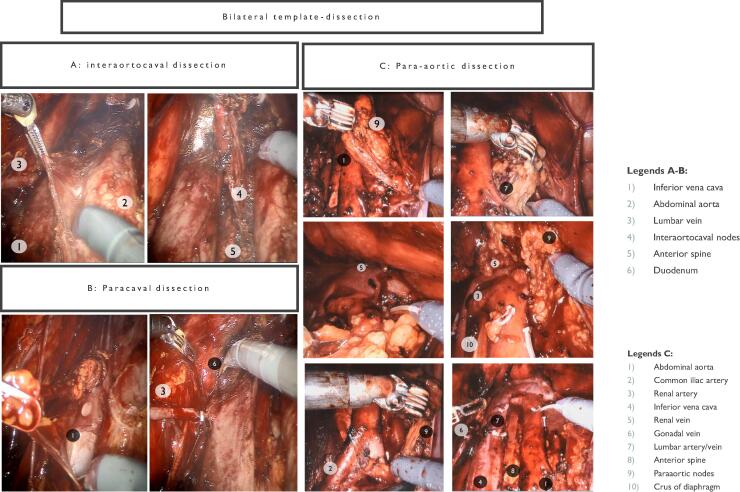
Bilateral template dissection. (A) Interaortocaval dissection. (B) Paracaval dissection. (C) Paraaortic dissection.

Table 1 lists the specialized equipment needed for the procedure. Fig. 1, Fig. 2 depict the operating room setups for unilateral and bilateral templates, respectively [24]. Fig. 3, Fig. 4, Fig. 5, Fig. 6, Fig. 7 illustrate the dissection limits for left unilateral, right unilateral, and bilateral template dissections [24]. RA-RPLND is utilized primarily for high-grade UTUC in the kidney, proximal ureter, and midureter. The unilateral template for RA-RPLND is shared across patients with testicular cancer and UTUC to optimize oncological outcomes and reduce procedural complexity.

#### 2.3.2 Left unilateral template

##### 2.3.2.1 Positioning

After general anesthesia induction, a Foley catheter is inserted. The patient is placed in a right lateral position, similar to the setup for a left robot-assisted nephroureterectomy (Fig. 1A). The operating table is flexed to improve access to the left abdominal region. Lateral supports stabilize the patient’s back, while the left leg is kept straight and the right leg flexed. Gel pads or other types of cushioning are applied at key pressure points to reduce the risk of neuropraxia. A nasogastric or orogastric tube is inserted intraoperatively to prevent gastric distension and facilitate safe port placement.

##### 2.3.2.2 Stages of the procedure

###### 2.3.2.2.1 Port placement

Ports are arranged linearly along the left linea semilunaris. Four 8-mm robotic ports are used, with a camera port inserted using the Hasson technique to establish a pneumoperitoneum at 12 mmHg. The assistant port is placed between the middle robotic ports, or in complex cases, it can be positioned along the midline between the superior robotic ports to improve access during renal vessel dissection (Fig. 1A). If using the da Vinci Si/Xi systems, adjustments such as moving the inferior robotic port laterally can minimize equipment clashes during the procedure.

###### 2.3.2.2.2 Robot docking

The robot is docked with the second-most superior port designated for the camera. For da Vinci Xi systems, targeting is performed on the para-aortic region inferior to the left renal vessels. The instruments used include monopolar scissors, fenestrated bipolar forceps, and Prograsp forceps. A 0° scope is preferred, with a 30° scope used for medial dissection.

###### 2.3.2.2.3 Descending colon mobilization

The line of Toldt is incised from the splenic flexure to the pelvis, mobilizing the descending colon medially. The spleen can also be mobilized to improve exposure to the left renal hilum. Gerota’s fascia is preserved initially to prevent inadvertent damage (Fig. 6).

###### 2.3.2.2.4 Left ureter identification

The left ureter is identified during colon mobilization, retracted laterally, and dissected from the renal pelvis superiorly to the crossing of the left common iliac artery inferiorly. Care is taken to avoid devascularization of the ureter, particularly in testicular cancer patients.

###### 2.3.2.2.5 Paraaortic dissection

Dissection begins where the ureter crosses the left common iliac artery. The tissue packet is isolated carefully from the psoas muscle, left common iliac artery, and aorta, progressing superiorly to the left renal vein. Posteriorly, lumbar vessels are clipped and divided to allow dissection. Care is taken to avoid injuring the inferior mesenteric artery.

###### 2.3.2.2.6 Interaortocaval dissection

Preaortic tissue is dissected medially off the anterior aspect of the aorta and into the interaortocaval groove. Dissection is extended superiorly to the left renal vein, ensuring that all dissection boundaries are complete.

###### 2.3.2.2.7 Specimen removal

Specimens are placed in Endocatch bags and extracted through extended incisions if necessary.

###### 2.3.2.2.8 Closure

Incisions are closed using absorbable sutures, with drains left only in cases involving UTUC.

This extended and detailed guide emphasizes critical aspects of the surgical technique, offering a robust framework for ensuring precision and minimizing complications.

#### 2.3.3 Right unilateral template

##### 2.3.3.1 Positioning

For the right unilateral template, the patient is positioned in the left lateral decubitus position (Fig. 1B), mirroring the left-sided approach.

##### 2.3.3.2 Stages of the procedure

###### 2.3.3.2.1 Port placement

Ports are placed along the right linea semilunaris. An additional 5-mm port can be inserted for liver retraction to improve exposure near the hepatic flexure.

###### 2.3.3.2.2 Robot docking

The docking setup mirrors the left template. Instruments include monopolar scissors, fenestrated bipolar forceps, and Prograsp forceps. A 30° scope is often necessary for medial dissection.

###### 2.3.3.2.3 Cecum and ascending colon mobilization

The line of Toldt is incised to mobilize the ascending colon and cecum medially. Gerota’s fascia is preserved, and blunt dissection is used to minimize trauma.

###### 2.3.3.2.4 Right ureter identification

The right ureter is identified during colon mobilization and retracted laterally. Dissection extends from the renal pelvis to the crossing of the right common iliac artery.

###### 2.3.3.2.5 Paracaval dissection

Starting at the point where the right ureter crosses the common iliac artery, the dissection progresses superiorly along the inferior vena cava (IVC). Lumbar veins are clipped and divided to mobilize the IVC for better exposure.

###### 2.3.3.2.6 Interaortocaval dissection

The anterior surface of the IVC and aorta is dissected using the split-and-roll technique. Lumbar veins are divided to facilitate retrocaval dissection.

#### 2.3.4 Bilateral template dissection

##### 2.3.4.1 Positioning

The patient is placed in a supine position, with arms tucked and gel pads placed for pressure point protection.

##### 2.3.4.2 Stages of the procedure

###### 2.3.4.2.1 Port placement

Ports are placed symmetrically in the left and right iliac fossae. Retraction sutures are used to elevate the posterior peritoneum for optimal visualization.

###### 2.3.4.2.2 Dissection

Retroperitoneal access is achieved by mobilizing the posterior peritoneum and duodenum. Dissections proceed along the IVC and aorta, and their respective borders, with particular attention to interaortocaval, paracaval, and para-aortic lymphatic packets (Fig. 7).

### 2.4 Statistical analyses

Qualitative data are presented as counts and percentages, and quantitative data as medians and interquartile ranges (IQRs).

## 3 Results

### 3.1 Testicular cancer cohort

Median age in the testicular cancer group was 32 (IQR 26–42) yr. The majority of patients (95%) were classified as American Society of Anesthesiologists (ASA) 1 and 2. Only 8% of patients had a Charlson Comorbidity Index of >2. Median body mass index (BMI) was 25.5 kg/m^2^. Median operative duration was 180 (IQR 150–206) min; 77% and 6% had previous chemotherapy (with the combination of bleomycin, etoposide, and cisplatin being the most frequent regimen [52%]) and abdominal surgery, respectively. Median estimated blood loss was 90 ml and transfusion rate was 5%. The three most common histopathological findings of the RPLND specimen were mature and differentiated teratoma (27% each), and seminoma (19%). Clinical nodal staging prior to chemotherapy and surgery revealed a relatively high proportion of node-negative disease (11% [7/64]) and cN1 (38% [24/64]). Clinical nodal stages 2 and 3 occurred in 42% (27/64) and 9% (6/64) of patients, respectively.

### 3.2 UTUC cohort

Median age in the UTUC group was 72 (IQR 67–77) yr. The majority of patients (78%) were classified as ASA 3, and 44% had a Charlson Comorbidity Index of >2. Median BMI was 26.7 kg/m^2^. Median operative duration was 223 (IQR 210–240) min; 6% had previous chemotherapy and 22% had previous abdominal surgery. Estimated median blood loss was 200 ml, and transfusion rate was 6%. Most of the tumors were located within the renal pelvis (44%).

### 3.3 Testicular cancer and UTUC cohort

Left, right, and bilateral template dissection was performed in 50, 24, and 22 procedures, respectively. Median lymph node counts were 16 and 12 in the testicular and UTUC cohorts, respectively. Two patients in the testicular cancer group undergoing salvage RPLND required open conversion for caval injury related to caval involvement of the nodal mass and conversion due to the inability to access the upper retroperitoneum safely with a supine approach because of the weight of the mesentery preventing adequate suspension of the mesentery.

The median length of stay of RA-RPLND patients was 1 (IQR 1–1) d for testicular cancer and 2.5 (IQR 2–4) d for UTUC patients. Major complications (Clavien-Dindo ≥3a) within 90 d occurred in six (9%) and four (13%) patients, respectively. Complications in the testicular cancer cohort include chylous ascites requiring percutaneous drainage (*n* = 3), port site hernia requiring laparoscopic repair (*n* = 1), and unplanned admission to the intensive care unit (ICU) immediately postoperatively (*n* = 2).

Major complications in the UTUC cohort include respiratory failure requiring ICU admission (*n* = 1), bleeding from a gastric ulcer (*n* = 1) and from gonadal artery requiring embolization (*n* = 1), and death due to myocardial infarction (*n* = 1).

Baseline characteristics, and peri- and postoperative data for both cohorts are summarized in Table 2, Table 3, Table 4.

**Table 2:** Comparison of peri- and postoperative data of 96 patients undergoing robotic retroperitoneal lymph node dissection for testicular cancer (*n* = 64) versus upper tract urothelial cancer (*n* = 32)

	Testicular cancer	Upper tract urothelial cancer
Age (yr), median (IQR)	32.1 (25.7–42.1)	72.1 (66.5–77.3)
Gender, *n* (%)		
Male	64 (100)	27 (84.4)
Female	0 (0)	5 (15.6)
BMI (kg/m^2^), median (IQR)	25.5 (22.5–30.7)	26.7 (24.2–32.6)
ASA, *n* (%)		
Class 1–2	61 (95)	7 (21.9)
Class 3	3 (5)	25 (78.1)
Charlson Comorbidity Index, *n* (%)		
≤2	59 (92)	18 (56.3)
>2	5 (8)	14 (43.7)
Previous chemotherapy, *n* (%)	49 (76.6)	2 (6.3)
Previous abdominal surgery, *n* (%)	4 (6)	7 (21.9)
Operative duration (min), median (IQR)	180 (300150–206)	222.5 (210–240)
Estimated blood loss (ml), median (IQR)	90 (50–150)	200 (100–300)
Transfusion, *n* (%)	3 (5)	2 (6)
Length of stay (d), median (IQR)	1 (1–1)	2.5 (2–4)
Conversions, *n* (%)	2 (3)	0 (0)
Major complications (Clavien-Dindo ≥3a), *n* (%)	6 (9)	4 (13)
Tumor laterality, *n* (%)		
Left	40 (63)	20 (63)
Right	24 (38)	12 (38)
Type of template, *n* (%)		
Unilateral left	29 (45)	20 (63)
Unilateral right	19 (30)	12 (38)
Bilateral	16 (25)	0 (0)
Lymph node count, median (IQR)	16 (9–25)	12 (7–17)

ASA = American Society of Anesthesiologists; BMI = body mass index; IQR = interquartile range.

**Table 3:** Peri- and postoperative data of 64 patients undergoing RA-RPLND for testicular cancer

	Testicular cancer
Tumor markers at the time of first diagnosis (testicular cancer), median (IQR)	
AFP	10 (2.9–155)
βHCG	4.95 (2–283)
LDH	290 (203–423)
Pathological T stage (testicular cancer), *n* (%)	
Embryonal carcinoma	30 (46.9)
Yolk sac	2 (3.1)
Teratoma	6 (9.4)
Seminoma	24 (37.5)
Leydig cell tumor	2 (3.1)
Clinical staging at the time of first diagnosis (testicular cancer)	
Clinical T stage, *n* (%)	
cT0	1 (1.6)
cT1	31 (48.4)
cT2	24 (37.5)
cT3	7 (10.9)
cT4	1 (1.6)
Clinical N stage at the time of orchidectomy, *n* (%)	
cN0	40 (62.2)
cN1	10 (15.5)
cN2	8 (12.5)
cN3	6 (9.4)
Clinical N stage prior to chemotherapy or primary RPLND, *n* (%)	
cN0	7 (10.9)
cN1	24 (37.5)
cN2	27 (42.1)
cN3	6 (9.4)
Clinical M stage, *n* (%)	
cM0	60 (93.8)
cM1	4 (6.2)
Previous (first-line) chemotherapy, *n* (%)	
BEP (3 cycles)	33 (51.6)
EP (4 cycles)	4 (6.2)
Other	12 (18.8)
None	15 (23.4)
Second-line chemotherapy, *n* (%)	
TIP	2 (3.1)
GAMEC	1 (1.6)
None	61 (95.3)
Indication of procedure, *n* (%)	
Primary RPLND	25 (39.1)
Postchemotherapy RPLND	29 (45.3)
RPLND for relapse tumor	9 (14.0)
Salvage RA-RPLND	1 (1.6)
Prognosis group (IGCCG), *n* (%)	
Good	51 (79.7)
Intermediate	8 (12.5)
Poor	3 (4.7)
None	2 (3.1)
Tumor markers prior to RA-RPLND, median (IQR)	
AFP (µg/l)	2.5 (2–4.8)
βHCG (IU/l)	0.2 (1–2)
LDH (U/l)	206 (166–238)
Histopathology of the RPLND specimen, *n* (%)	
Embryonal carcinoma	7 (10.9)
Leydig cell carcinoma	2 (3.1)
Mature teratoma	17 (26.6)
Necrosis	7 (10.9)
Sarcomatoid carcinoma	1 (1.6)
Seminoma	12 (18.7)
Differentiated teratoma	17 (26.6)
Yolk sac tumor	1 (1.6)
Size of main mass on imaging (mm), median (IQR)	
Prior to chemotherapy	22 (18–30)
Prior to RPLND	21.5 (16–35)
Gonadal vein involvement, *n* (%)	0 (0)
Positive surgical margin, *n* (%)	0 (0)
Secondary metastasis, *n* (%)	
Abdominal lymph nodes	57 (89.0)
Abdominal lymph nodes and lungs	1 (1.6)
Abdominal lymph nodes and lungs and brain	1 (1.6)
Lungs and brain	1 (1.6)
None	4 (6.2)

AFP = alpha-fetoprotein; BEP = bleomycin, etoposide, and cisplatin; EP = etoposide and cisplatin; GAMEC = actinomycin-D, methotrexate, cisplatin, and etoposide; HCG = human chorionic gonadotropin; IGCCG = International Germ Cell Cancer Collaborative Group; IQR = interquartile range; LDH = lactate dehydrogenase; RA-RPLND = robot-assisted retroperitoneal lymph node dissection; RPLND = retroperitoneal lymph node dissection; TIP, = paclitaxel, ifosfamide, and cisplatin.

**Table 4:** Peri- and postoperative data of 32 patients undergoing RA nephroureterectomy and RPLND for upper tract urothelial cancer

	Upper tract urothelial cancer
Hemoglobin (g/l), median (IQR)	135.5 (120–145)
Serum creatinine (umol/l), median (IQR)	101 (83–134)
eGFR preoperatively (ml/min), median (IQR)	62.5 (43–71)
Size of tumor on imaging (cm), median (IQR)	4.5 (3.1–8.6)
Diagnosis with ureterorenoscopy, ureteric washing, and/or cytology, *n* (%)	26 (91)
Preoperative biopsy, *n* (%)	
High grade	7 (21.9)
Low grade	7 (21.9)
Equivocal	6 (18.7)
None	12 (37.5)
Preoperative cytology (ureteric washing), *n* (%)	
Positive (for high grade)	8 (25)
Negative (for high grade)	6 (18.7)
Equivocal	8 (25)
None	10 (31.3)
Diagnosis with imaging only, *n* (%)	6 (19)
Neoadjuvant chemotherapy, *n* (%)	2 (6.3)
Clinical staging, *n* (%)	
T stage	
cTa	6 (18.8)
mortcT1	11 (34.4)
cT2	6 (18.8)
cT3/4	5 (15.6)
cTX	4 (12.5)
N stage	
cN0	24 (75)
cN1	5 (15.6)
cN2	3 (9.4)
M stage	
cM0	32 (100)
cM1	0 (0)
Histopathology	
Size of tumor (mm), median (IQR)	4.5 (2.9–6.3)
T stage, *n* (%)	
pTa	12 (37.5)
pT1–2	7 (21.9)
pT3–4	13 (40.6)
N stage, *n* (%)	
pN0	26 (81.2)
pN1	4 (12.5)
pN2	2 (6.3)
Grade, *n* (%)	
Low grade	5 (15.6)
High grade	27 (84.4)
Concomitant carcinoma in situ, *n* (%)	9 (28.1)
Positive surgical margins, *n* (%)	2 (6.3)
Tumor location, *n* (%)	
Renal pelvis	14 (43.8)
Proximal ureter	8 (25)
Midureter	10 (31.2)

eGFR = estimated glomerular filtration rate; IQR = interquartile range; RA = robot assisted; RPLND = retroperitoneal lymph node dissection.

Two patients died within 90 d after RA-RPLND for UTUC; one due to an acute myocardial infarction and the other due to progressive disease. Six patients (19%) with UTUC died due to progressive disease within a median follow-up of 38 (range 4–66) mo, whereas all patients with testicular cancer were still alive after a median follow-up of 46 (range 1–97) mo. Overall and cancer-specific survival rates at the end of follow-up were 78% and 69% in patients with UTUC, and 100% and 100% in patients with testicular cancer, respectively. No retroperitoneal recurrences occurred in either cohort until the end of follow-up.

## 4 Discussion

RA-RPLND for testicular cancer and UTUC is a safe procedure and offers comparable morbidity to O-RPLND, with potential benefits including reduced length of stay, reduced blood loss, and decreased operative duration. Furthermore, there may be benefits in anejaculation rates with the robot-assisted approach.

There have been several series that have reported their results of primary and postchemotherapy RA-RPLND for testicular cancer. The role of lymph node yield as a quality control measure is of less value in the postchemotherapy RPLND setting, as many of the residual masses will consist of a conglomerate of fibrotic and necrotic lymph nodes that will be counted as a single lymph node only [24], [25]. Lymph node yields are also highly susceptible to variability due to processing procedures [26]. Hence, the lymph node yields may be higher in series that included a higher proportion of primary RPLND procedures and in those with bilateral template dissections.

There have been several studies that have analyzed the suitability of unilateral templates in the postchemotherapy setting. Concerns have been raised regarding the oncological outcomes and risk of recurrence with such an approach. However, long-term follow-up has shown that oncological outcomes may not be affected [25], [27]. In particular, recurrence in the contralateral side of the retroperitoneum within the field of a bilateral template was not significantly different.

Owing to the similarity of lymph node metastasis patterns for renal pelvis and proximal ureteral tumors, and evidence for retroperitoneal lymphatic spread in midureteral tumors [9], [20], we adhere to a uniform ipsilateral dissection template. It has been shown that a right-sided unilateral template involving dissection of the hilum and paracaval, retrocaval, and interaortocaval regions would capture nearly all (96%) lymph node metastases. Likewise, for the left side, hilar para-aortic and interaortocaval dissection would capture 90% of lymph node metastases [9].

Even though a recent meta-analysis on the effect of LND on the outcomes of UTUC showed improved survival, particularly for locally advanced tumors, robust data on oncological outcomes remains sparse [11], [15]. Therefore, the role and extent of RPLND at the time of radical nephroureterectomy remains controversial. However, we advocate considering template-based RPLND because it provides more accurate staging even if it might not confer a direct survival benefit.

To our knowledge, this is the first study that comments on RA-RPLND from a more comprehensive combined testicular cancer and UTUC point of view, and might be useful for both preoperative preparations by the performing surgeon and patient counseling peri- and postoperatively.

When considering more recent studies analyzing the open approach, McFadden et al [28] have demonstrated that primary O-RPLND has an acceptable morbidity profile. Similarly, Syan-Bhanvadia et al [29] have shown that the open midline extraperitoneal approach can be performed safely.

As such, O-RPLND is safe and effective at select tertiary centers especially when combined with the Enhanced Recovery After Surgery pathway, even in postchemotherapy patients, and with higher extent of disease [30].

RA-RPLND remains a technically challenging operation and is primarily used in select high-volume centers owing to the steep learning curve and its technical complexity. Therefore, a limitation of this work is that results cannot necessarily be reproduced by other surgeons who perform robot-assisted surgery.

Similarly, extensive disease and tissue changes after chemotherapy may require adjunct procedures during RPLND, which may implicate particularly challenging situations, especially when attempting a robot-assisted approach. This in turn implicates a relevant limitation in the use of the robot for some complex postchemotherapy cases. As such, while preoperative imaging can help predict vascular involvement in chemotherapy-naïve patients in particular, encasement of major vessels in the postchemotherapy setting may be encountered only intraoperatively.

For this reason, to safely perform RA-RPLND, surgical expertise must be available to perform an O-RPLND in the event of conversion (eg, for vascular control and repair). As such, patients in the testicular cancer cohort have been selected very well, which is a relevant limitation of the study.

When considering RA-RPLND in testicular cancer and taking the burden of disease in the RA-RPLND cohort into account, smaller size of retroperitoneal masses as well as the lesser extent and distribution of retroperitoneal disease are factors to consider to ensure a safe procedure, whereas encasement of great vessels and involvement of multiple viscera are absolute contraindications to perform RA-RPLND. In accordance with these guiding principles, the median size of the retroperitoneal masses in our testicular cancer cohort was <3 cm, and the proportion of patients with clinically node-negative and node-positive disease with mass size ≤2 cm was relatively high. Of note, a relevant number of patients had been staged as cN0 after orchidectomy and then relapsed on surveillance and proceeded to chemotherapy and surgery. Furthermore, clinical N stage may be underestimated in a number of cases (i.e., some patients with a cN0 status may actually have cN1, and some of the cN1 patients may actually be cN2 patients), which is a limitation of the study.

Predominance of disease that is posterior, especially retroaortic or retrocaval, will be more difficult to dissect, and any vascular injury will be more difficult to repair. Masses that have shrunk significantly after chemotherapy tend to be more fibrotic and hence can be more challenging to dissect than larger cystic masses. Dissection is also more difficult in patients who have had multiple lines of chemotherapy.

The learning curve for RA-RPLND is difficult to assess given the small series, including our own, available. Cases were selected on a case-by-case basis, and hence there was no absolute restriction or indication based on size for performing a robotic approach. The main concern with such surgery is vascular injury, and hence the relationship of the residual disease with the great vessels was perhaps more crucial than the size of the mass. Inevitably, this introduces a selection bias in the robotic group as the alternative to perform O-RPLND was available.

## 5 Conclusions

In expert hands, RA-RPLND for testicular cancer and UTUC is a safe procedure, and therefore, application of this approach to various surgeries should be considered in selected patients.

Ultimately, more studies with longer-term follow-up comparing RA-RPLND with O-RPLND directly are needed to effectively compare clinical efficacy in terms of oncological outcomes relative to complications and to achieve agreement on the extent of the LND template, particularly in UTUC patients.

  ***Author contributions:*** Marc A. Furrer had full access to all the data in the study and takes responsibility for the integrity of the data and the accuracy of the data analysis.

  *Study concept and design*: Furrer, Thomas.

*Acquisition of data*: Thomas.

*Analysis and interpretation of data*: Furrer, Thomas.

*Drafting of the manuscript*: Furrer, Thomas.

*Critical revision of the manuscript for important intellectual content*: Furrer, Thomas.

*Statistical analysis*: Furrer.

*Obtaining funding*: None.

*Administrative, technical, or material support*: None.

*Supervision*: Thomas.

*Other*: None.

  ***Financial disclosures:*** Marc A. Furrer certifies that all conflicts of interest, including specific financial interests and relationships and affiliations relevant to the subject matter or materials discussed in the manuscript (eg, employment/affiliation, grants or funding, consultancies, honoraria, stock ownership or options, expert testimony, royalties, or patents filed, received, or pending), are the following: None.

  ***Funding/Support and role of the sponsor:*** None.
