# Supplementary material for: Robot-assisted Laparoscopic Retroperitoneal Lymph Node Dissection for Testicular and Upper Tract Urothelial Cancer—Surgical Technique and Outcomes of a Single-surgeon Series
Source: Eur Urol Open Sci. 2025 Apr 15;75:120–32. doi: 10.1016/j.euros.2025.03.015 (PMC12032178; doi:10.1016/j.euros.2025.03.015)
Supplement: Supplementary Data 1 [file mmc1.docx]

**Supplemental Material 1:**

- 1. Indications and Contraindications for Robot-Assisted RPLND
     1. Testicular cancer

There are some geographical variations in the indications for RPLND for testicular-cancer between North-America and Europe, particularly for primary-RPLND in non-seminomatous germ-cell-tumour. In North-America, primary-RPLND is offered in high-risk Stage-1 non-seminomatous germ-cell-tumour and Stage-2A and 2B-non-seminomatous germ-cell-tumour. Primary-RPLND is increasingly being recommended for Stage-2A-disease in Europe. Universally, RPLND is recommended for residual masses >1cm after chemotherapy where tumour-markers have normalised or in the event of retroperitoneal-relapse with normal markers. RPLND is also being utilised in the trial setting for Stage-2-seminoma. There are no absolute indications for a robot-assisted approach being considered.

Table 1a lists factors that were considered in determining whether robot-assisted RPLND may be feasible. As most robot-assisted approaches only allow dissection from a limited number of directions, the size of the mass should be small enough to enable adequate exposure of the mass. A threshold of 5cm could be used as a guideline from this perspective. The relationship of the disease to the great vessels is perhaps the most important aspect. Predominance of disease that is posterior, especially retroaortic or retrocaval, will be more difficult to dissect and any vascular injury will be more difficult to repair. Masses that have shrunk significantly after chemotherapy tend to be more fibrotic and hence can be more challenging to dissect than larger cystic masses. Dissection is also more difficult in patients who have had multiple lines of chemotherapy.

Table 1b and 1d list absolute contraindications to robot-assisted RPLND. For robot-assisted RPLND, there must be surgical expertise available to perform an open RPLND in the event of conversion and also vascular surgical expertise for vascular control and repair. Table 1c lists advantages and disadvantages of the robot-assisted approach as compared to the open approach.

- - 1. Upper tract urothelial cancer

Invasive or large, and primarily metastasized UTUC (T3/T4 and/or N+/M+) were considered contraindications for the robotic-approach as the outcome is worse compared to an open-approach whereas open, laparoscopic and robotic-approaches have similar oncological-outcomes for organ-confined UTUC(15, 24), see also Table 1d.

Based on available data regarding patterns of lymphatic-metastases in UTUC we perform a unilateral retroperitoneal lymph-node template-dissection in all patients with renal-pelvis and proximal to mid ureteral-tumor. For mid ureteral-tumors we additionally perform an extended pelvic LND described by Zehnder et al(25).

- 1. Pre-Operative Assessment

All cases considered for RPLND should be discussed in a multidisciplinary setting. If surgery is recommended, surgical planning should begin with extensive review of the patient’s most recent cross-sectional imaging and any previous imaging, particularly, pre-chemotherapy imaging.

In testicular-cancer patients, a decision regarding unilateral or bilateral template-dissection should be made based on institutional policy as there remains significant conjecture regarding suitability of unilateral-templates, particularly in the post-chemotherapy setting. The relationship of the disease to the renal-vessels and ureters should be assessed. Pre-operative ureteric-stenting may be helpful if the ureters appear densely adherent to the retroperitoneal-disease. Assessment of the proximity of retroperitoneal-disease to the duodenum and great vessels should be made to determine if specialty assistance from a vascular-surgeon or general-surgeon may be required.

Routine blood-tests should be performed pre-operatively to assess the patient’s baseline renal-function and also to assess whether haematological parameters have returned to the normal range after previous chemotherapy. If bleomycin was given as part of the chemotherapy-regimen, respiratory assessment should be made to determine if additional measures are required peri-operatively such as pre-operative steroids and anaesthetic-management of intra-operative ventilation. Blood-grouping should be done to ensure there is adequate facility for transfusion in the event of unexpected haemorrhage.

For UTUC, post-operative renal impairment due to nephroureterectomy should be considered and the patient appropriately counselled. Nephrology input should be sought as needed.

- 1. Post-Operative Instructions

Patients should be given deep venous-thromboprophylaxis as soon as possible after surgery and is recommended for 28 days. Furthermore, patients should be encouraged to mobilise immediately. Simple oral-analgesia is recommended to avoid any opioid-related issues post-operatively. Light-diet can be commenced immediately after surgery and progressed rapidly. If there is concern regarding chylous-ascites developing, the patient can be placed on a medium-chain-fatty-acid-diet for a week to minimise its occurrence. Blood-tests can be performed the following day to ensure no significant haemorrhage has occurred and confirm renal-function was unaffected. Patients can usually be discharged the following day if their pain is controlled, they are tolerating diet and are mobilising.
