# Supplementary material for: Robot-assisted Laparoscopic Retroperitoneal Lymph Node Dissection for Testicular and Upper Tract Urothelial Cancer—Surgical Technique and Outcomes of a Single-surgeon Series
Source: Eur Urol Open Sci. 2025 Apr 15;75:120–32. doi: 10.1016/j.euros.2025.03.015 (PMC12032178; doi:10.1016/j.euros.2025.03.015)
Supplement: Supplementary Data 2 [file mmc2.docx]

# Supplementary Material 2

**Surgical technique**

*Theatre and port setup*

Table-1e lists the special equipment required. Figures-1 and- 2 show the operating room-setups for unilateral and bilateral templates respectively.

Figures 4-6A show the dissection limits for left-unilateral, right-unilateral and bilateral template-dissections. We performed RA-RPLND for high grade UTUC in the kidney, proximal and mid-ureter. The same unilateral-template is performed for patients with testicular-cancer and UTUC.

1. **Left Unilateral Template**
   1. *Positioning*

After induction of general anaesthesia, a Foley catheter is placed. The patient is positioned in the right-lateral position - similar to a left robot-assisted nephrectomy (Figure 1A). The bed is flexed to open up the left lateral aspect of the abdomen. Lateral supports are placed to support the patient’s back. The arms are flexed at the elbows with the shoulder at 90-degrees. The left leg should be straight with the right leg flexed. Gel-pads or supports should be placed at the right ankle and right knee to minimise neuropraxias. A nasogastric or orogastric-tube is place intraoperatively to decrease any gastric distension for safe port placement.

Stages of the Procedure

- 1. *Port Placement*

A linear configuration of ports is used with the four 8mm robotic ports placed along the left linea semilunaris. The camera-port is inserted using a Hasson technique and pneumoperitoneum is then created at 12mmHg. The remaining ports are inserted under vision. The 12mm assistant-port is placed in between the middle two ports. If a difficult dissection on the renal vessels is anticipated, it may be worth considering placing the assistant-port in the midline between the superior two ports (Figure 1A). For the da Vinci Si/X, it may be worth moving the inferior 8mm robotic port 3cm laterally to minimise clashes.

- 1. *Robot Docking*

The robot is docked with the second-most superior robotic port being the camera-port. For the da Vinci Xi, targeting is performed with the target being the predicted para-aortic region inferior to the left renal vessels. The remaining ports are then docked (Figure 1C).

A fenestrated bipolar forceps is placed in the superior robotic port, monopolar scissors in the second-most inferior robotic port and a prograsp forceps in the inferior robotic port. A 0-degree scope is used however a 30-degree scope may be necessary for the medial aspects of dissection.

- 1. ***Dissection***
     1. *Descending Colon Mobilisation*

The descending colon is mobilised by an incision along the line of Toldt from the splenic flexure to the left aspect of the pelvis inferior to the iliacvessels (Figure 3A). Gerota’s-fascia over the left kidney should be left intact at this time. The spleen can be mobilised more medially by extending the incision in the posterior-peritoneum inferior to-the spleen toward the diaphragm. This should enable appropriate exposure of the left renal hilum (Figure 3B).

- - 1. *Left Ureter Identification*

The left-ureter is identified during mobilisation of the descending colon and then retracted laterally using the prograsp forceps. This should allow exposure of the left psoas muscle posteriorly. Too much tension on the left-ureter should be avoided to prevent devascularisation of its tenuous blood supply in testicular cancer cases. The medial aspect of the left-ureter can then be dissected to the renal pelvis superiorly and to the crossing of the left common iliac artery inferiorly – this represents the lateral border of the template. The left-gonadal vein will be lateral to the ureter in the inferior aspect of this region and will be more medial to the ureter where it joins the left-renal vein (Figures 3C-D).

- - 1. *Para-Aortic Dissection*

The template dissection is commenced at the point where the left-ureter crosses the left-common iliac-artery (Figure 4A). A tissue packet is isolated from these structures and dissection continues superiorly by dissecting the tissue packet off the psoas muscle posteriorly and the left-common iliac artery medially (Figure 4B). As the dissection progresses, the tissue packet is dissected laterally off the aorta. The inferior mesenteric artery will arise from the anterior aspect of the aorta and will pass to the patient’s right due to the descending colon having been mobilised medially. Posteromedially, lumbar arteries and veins may be encountered and should be clipped with hem-o-loks or clips and divided. Dissection proceeds along the aorta until the left-renal vein is reached. The tissue packet is then dissected inferiorly off the left-renal-vein (Figure 4C). The dissection should then be completed with the dissection along the left-renal vein joining the previous dissection of the left-ureter. For testicular cancer cases, the left-gonadal vein should be clipped and divided as it enters the left-renalvein. The left-gonadal vein should then be dissected to the left deep inguinal-ring and clipped and divided to enable its complete excision (Figure 4A). The specimens can then be placed in an Endocatch bag.

- - 1. *Interaortocaval dissection*

Pre-aortic tissue can be dissected off the anterior aspect of the aorta superior to the origin of the inferior mesenteric artery. This tissue packet can be then dissected further medially off the right-aspect of the aorta and into the interaortocaval groove. The anterior spinous ligament will be encountered posteriorly (Figure 4D). The tissue packet should then be retracted to the left. This should enable the left border of the IVC to be seen and therefore allow dissection of the tissue-packet away from the IVC. The left-and right-borders of the dissection are then completed. Completion of the dissection occurs with the tissue-packed being dissected off the left-renal vein superiorly to complete the dissection. The specimen can then be placed in an Endocatch-bag.

1. **Right-Unilateral Template**
   1. *Positioning*

The patient is positioned in the left-lateral position mirroring the positioning described previously for a left-unilateral template (Figure 1B).

Stages of the Procedure

- 1. *Port Placement*

A linear configuration of ports is used with the four 8mm robotic-ports placed along the right-linea semilunaris when using the da Vinci Xi. The port placement mirrors the placement for a left-unilateral template described previously.-A 5mm-port may be inserted just inferior to the xiphisternum to allow a ratcheted forcep to act as a liver-retractor (Figure 1B).

- 1. *Robot Docking*

The robot is again docked with the second-most superior robotic port being the camera port. For the da Vinci Xi, targeting is performed with the target being the IVC at the level of the renal veins. The remaining ports are then docked-(Figure 1C). A monopolar scissors is placed in the superior robotic port, fenestrated bipolar forceps in the second-most inferior robotic port and a prograsp forceps in the inferior robotic port.-A 0-degree scope is used however a 30-degree scope is often necessary for the medial aspects of dissection.

- 1. ***Dissection***
     1. *Caecum and Ascending Colon Mobilisation*

The ascending-colon is mobilised by an incision along the line of Toldt from the hepatic flexure to the right-aspect of the pelvis inferolateral to the caecum. Gerota’s fascia over the right-kidney should be left intact at this time. The ascending colon and caecum are mobilised medially using predominantly blunt dissection (Figure 5B). The second-part of the duodenum will then be encountered in the superior aspect of the dissection.

- - 1. *Mobilisation of the Duodenum*

The second-part of the duodenum is mobilised using a combination of sharp and blunt dissection using Kocher’s manoeuvre-(Figure 5C). The third-part of the duodenum should also be mobilised as much as possible to enable adequate exposure of the left-aspect of the IVC.

- - 1. *Right Ureter Identification*

The right-ureter is identified during mobilisation of the ascending colon and then retracted laterally using the prograsps (Figure-3C-D). The medial aspect of -right-ureter can then be dissected to the renal pelvis superiorly and to the crossing of the right-common iliac artery inferiorly – this represents the lateral-border of the template. The right-gonadal vein will lie medial to the right-ureter in this area of dissection and will join the IVC in contrast to the left-gonadal vein hence it can be reflected medially.

- - 1. *Paracaval Dissection*

This dissection is analagous to the para-aortic dissection in a left-unilateral template. The template dissection is commenced at the point where the right-ureter crosses the right-common iliac artery. A tissue packet is isolated from these structures and dissection continues superiorly by dissecting the tissue packet off the psoas muscle posteriorly and the right-common iliac artery medially. As the dissection progresses, a split-and-roll technique is performed over the anterior aspect of the IVC with the tissue packet dissected laterally off the IVC. Posteromedially, lumbar veins should be clipped and divided to enable adequate mobilisation of the IVC which is particularly useful for the interaortocaval dissection (Figure 5D). Dissection proceeds along the IVC until the right-renal vein is reached. The tissue packet is then dissected inferiorly off the right-renal vein and excised off the right-ureter. The right-gonadal vein which was previously reflected medially can then be clipped and divided at the level of the IVC and also at the right deep inguinal ring for excision. The specimen can then be placed in an Endocatch bag.

- - 1. *Interaortocaval dissection*

Precaval tissue can be dissected off the anterior aspect of the IVC where the split-and-roll was performed. This tissue packet can be then dissected further medially off the left-aspect of the IVC into the interaortocaval groove. The IVC can be reflected laterally with the prograsp or the assistant’s suction-device to enable the left-aspect of the IVC to be completely dissected and also to enable retrocaval tissue to be dissected. The anterior spinous ligament will be encountered posteriorly (Figure-5E). Once the IVC is freed, the tissue packet should then be retracted to the right. This should enable the right-border of the aorta to be seen and therefore allow dissection of the tissue packet away from the aorta and right common iliac artery as it passes over the IVC. The left-and right-borders of the dissection are then completed. Completion of the dissection occurs with the tissue packet being dissected off the left renal vein superiorly to complete the dissection. The specimen can then be placed in an Endocatch-bag.

- - 1. *Closure*

The specimens are usually quite small and can be removed though a small extension of the assistant port for testicular cancer cases or through the extraction nephroureterectomy wound for UTUC cases. The rectus-sheath for this incision and the camera-port are closed with 1-PDS. 3-0 Vicryl-Rapide is used for skin closure. No drain is left for testicular cancer cases. A 15Fr-Blake-drain is left in the pelvis for UTUC-cases given the closure of the bladder-cuff-excision.

1. **Bilateral Template Dissection**

This is likely to be utilized for testicular cancer given only unilateral templates are recommended for UTUC.

- 1. *Positioning*

The patient is placed supine with arms tucked. Gel-pads should be placed under the heels.

Stages of the Procedure

- 1. *Port Placement*

An 8mm camera-port is inserted via an open Hasson-technique in the midline 4cm superior to the pubis. Pneumoperitoneum is created at 12mmHg. An 8mm robotic-port is placed in the left-iliac-fossa approximately 8cm lateral and 2cm superior to the camera-port. Another 8mm robotic-port is inserted 8cm lateral and 2cm superior to the previously described left iliac fossa robotic port (Figure-2A). Clearance from the left-anterior superior iliac spine should be checked to minimise any potential restriction in range of movement. The ports in the right-iliac-fossa mirror those inserted in the left-iliac fossa with the more medial port being a 12mm assistant-port and the more lateral port being an 8mm robotic-port.

- 1. *Robot Docking*

The patient is placed in steep Trendelenburg-position before docking the robot. This enables the small bowel to fall superiorly as much as possible and enable visualisation of the posterior peritoneum. The robot is docked with the ports pointing towards the head (Figure-2B). A fenestrated bipolar forceps is place in the right lateral iliac fossa port. Monopolar-scissors are placed in the medial left-iliac-fossa port and a prograsps forceps placed in the lateral left iliac-fossa port. The ports should be pulled up anteriorly on the anterior abdominal wall to increase the working space

- 1. ***Dissection***
     1. *Retroperitoneal Access and Exposure*

The small bowel is pushed as much as possible into the left-and right-upper-quadrants. The right-ureter is then identified as it crosses the right-common iliac artery (Figure 6B). The incision is commenced at this point and continued diagonally - superiorly and to the patient’s left over the anterior aspect of the right-common iliac artery and aorta towards the ligament of Treitz (Figure-6C). The superior leaf of posterior peritoneum is then lifted anteriorly and the pneumoperitoneum is used in combination with blunt dissection to lift the posterior peritoneum off the IVC and aorta. The third-part of the duodenum is then reached and dissected anteriorly off the IVC and aorta until the left-renal vein is identified (Figure-6D).

Retraction sutures are then used to hold-up the posterior peritoneum and enable dissection over the great vessels. 1-Prolene on a straight needle is passed through the anterior abdominal wall in the right upper quadrant and passed through the right-superior aspect of the posterior peritoneum that has been dissected and then passed back through the abdominal wall. The suture is then pulled up externally and an artery-clip is placed flush against the skin at the appropriate level of retraction. A second 1-Prolene on a straight needle is then passed through the anterior abdominal wall in the left-upper quadrant and passed through the posterior peritoneum near the ligament of Treitz and passed back through the anterior abdominal wall and secured with an artery-clip (Figure-6D). These retraction or “hammock”-sutures should enable a good view of the IVC and aorta to the level of the left-renal vein with the superior mesenteric artery in the distance superiorly. The prograsp forceps can then be used to retract the third-part of the duodenum anteriorly to create more space.

- - 1. *Interaortocaval Dissection*

Dissection is commenced over the right common iliac artery and carries on superiorly by performing a split-and-roll of tissue over the anterior aspect of the aorta. The interaortocaval tissue can then be retracted to the right, away from the aorta. A similar split-and-roll is performed over the anterior aspect of the IVC and continued to the level of the left-renal-vein. The interaortocaval tissue is reflected to the left away from IVC. Lumbar-veins in the interaortocaval groove should be clipped with hem-o-loks and divided to enable mobility of the IVC. The medial and lateral dissection of the interaortocaval packet will reach the anterior spinous-ligament posteriorly (Figure-7A). The packet can then be dissected off the anterior spinous ligament with the dissection carried superiorly to the level of the left-renal vein and right renal artery. Care should be taken at the superior aspect of this dissection to ensure that these structures are preserved. Large lymphatic vessels at the level of the renal vessels should be clipped to minimise the risk of chylous ascites. The interaortocaval packet can then be excised.

- - 1. *Paracaval Dissection*

Paracaval dissection is also commenced at the point of the right-ureter crossing the right-common iliac artery. The right-ureter is completely dissected laterally to the level of the right-renal hilum. The paracaval tissue packet is dissected off the lateral aspect of the IVC to the level of the right-renal vein. The IVC can be retracted medially to enable retrocaval tissue to be incorporated in the packet. Lumbar veins arising of the lateral aspect of the IVC can be clipped with hem-o-loks to further enable this dissection. The right-gonadal vein can be clipped with hem-o-loks and divided at the IVC and right-inguinal ring for a right-sided primary testicular tumour (Figure-7B).

- - 1. *Para-Aortic Dissection*

The para-aortic dissection is commenced by identifying the left-ureter and reflecting it lateral to complete the lateral dissection of the paraaortic packet. The left-ureter is dissected from its crossing over the left-common iliac artery to the left renal hilum. The para-aortic tissue packet is then dissected laterally of the lateral aspect of the aorta until the left-psoas muscle is reached posteriorly. The para-aortic packet is then dissected off the left psoas muscle to the left-renal vein and excised (Figure 7C). The inferior mesenteric artery is identified as it arises from the aorta and passes inferolaterally to the patient’s left. It can usually be preserved with the potential need to split the para-aortic tissue in superior and inferior packets over the inferior mesenteric-artery. If a mass is densely adherent, division of the inferior mesenteric artery should be considered. The left-gonadal vein can be clipped with hem-o-loks and divided at the left-renal-vein and left deep inguinal-ring at this time for left-sided testicular tumours.

The specimens should be placed in Endocatch bags and either brought through the assistant port-incision if small or by extension of the suprapubic camera-port-incision if larger.
